# Supplementary material for: On-Chip Quantum Sensing of Kondo Spins in a High-Mobility Quasi-One-Dimensional Nanoconstriction
Source: Nano Lett. 2025 May 1;25(19):7740–7. doi: 10.1021/acs.nanolett.5c00560 (PMC12082700; doi:10.1021/acs.nanolett.5c00560)
Supplement: Supplementary file 1 — nl5c00560_si_001.pdf [file nl5c00560_si_001.pdf]

Supporting Information

On-chip quantum sensing of Kondo spins in a high-mobility quasi-one-dimensional nanoconstriction

Shun-Tsung Lo[1,2*], Che-Cheng Wang[2], Sheng-Chin Ho[2], Jun-Hao Chang[2], Ming-Wei Chen[2], G. L. Creeth[3], L. W. Smith[2], Shih-Hsiang Chao[2], Yu-Chiang Hsieh[2], Pei-Tzu Wu[1], Yi-Cheng Wu[1], Chi-Te Liang[4], M. Pepper[3], J. P. Griffiths[5], I. Farrer[5,6], G. A. C. Jones[5], D. A. Ritchie[5], & Tse-Ming Chen[2,7*]

[1]*Department of Electrophysics and Center for Emergent Functional Matter Science, National Yang Ming Chiao Tung University, Hsinchu 300, Taiwan*

[2]*Department of Physics, National Cheng Kung University, Tainan 701, Taiwan*

[3]*Department of Electronic and Electrical Engineering, University College London, London WC1E 7JE, United Kingdom*

[4]*Department of Physics, National Taiwan University, Taipei 106, Taiwan*

[5]*Cavendish Laboratory, J J Thomson Avenue, Cambridge CB3 0HE, United Kingdom*

[6]*Department of Electronic and Electrical Engineering, University of Sheffield, Mappin Street, Sheffield S1 3JD, United Kingdom*

[7]*Center for Quantum Frontiers of Research & Technology (QFort), National Cheng Kung University, Tainan 701, Taiwan*

*To whom correspondence should be addressed;

E-mail: stlo@nycu.edu.tw; tmchen@phys.ncku.edu.tw.

Supporting Note 1. Experimental methods

Device fabrication. Devices are fabricated on the GaAs/AlGaAs heterostructure containing a two-dimensional electron gas (2DEG) beneath the surface. The following layer sequence is grown by molecular beam epitaxy: 1μm GaAs (buffer), 40 nm AlGaAs (spacer), 40 nm AlGaAs (Si-doped), and 10 nm GaAs (cap) for test devices T1 and T2; 1μm GaAs (buffer), 75 nm AlGaAs (spacer), 200 nm AlGaAs (Si-doped), and 10 nm GaAs (cap) for devices A and B. The surface gates for the formation of quantum point contact (QPC), quantum dot (QD), and electronic resonator (ER) are subsequently defined by electron-beam lithography and thermal evaporation of Ti/Au. For device B, the ER finger gates are deposited on top of the QPC split gates and they are electrically isolated by a 45-nm-thick SiO_2 insulator. The 2DEG has an electron carrier density and mobility of 2.0×10^{11} (3.3×10^{11}) cm^{-2} and 3×10^{6} (5×10^{6}) cm^{2}V^{-1}s^{-1}, respectively, giving a mean free path of 20 (50) µm, before (after) full illumination for devices T1 and T2. The 2DEG has an electron carrier density and mobility of 8.6×10^{10} (1.4×10^{11}) cm^{-2} and 4×10^{6} (6×10^{6}) cm^{2}V^{-1}s^{-1}, respectively, giving a mean free path of 20 (40) µm, before (after) full illumination for devices A and B.

Quantum transport measurements. Measurements on partially illuminated devices were performed in a dilution refrigerator at a base temperature of 17 mK unless otherwise stated. The differential conductance of QPC was monitored in linear response to an excitation source of a 77 Hz ac voltage of 50 µV plus a dc source-drain bias voltage V_{sd}. Conductance data collected at $V_{sd}=0$ and as a function of V_{sd} are referred to as linear and nonlinear conductance, respectively. In devices T1 and T2, all the gates are energized in the studies and the conductance of QPC and QD were simultaneously monitored using the same source electrode but different drain ones. The

QPC nonlinear conductance was obtained by providing a dc bias to the QPC drain (see the insets of Supporting Figs. S1a and S2a for the device gate patterns). An in-plane magnetic field is applied to the device for magnetotransport measurements.

Supporting Note 2. Discussion on approaches for detecting Kondo effects in QDs and QPCs

The two-impurity Kondo effect is a subtly different phenomenon beyond the standard Kondo screening. The isolated magnetic impurities not only interact with the sea of itinerant electrons but also with one another via their exchange interactions. This complex many-body phenomenon is usually investigated in semiconductor double QDs and contains rich physics[1–5], where understanding these unconventional Kondo correlations can help address many strongly correlated multiple-impurity systems such as high-temperature superconductors and heavy fermion materials[6–11]. A split ZBA is the key signature of two-impurity Kondo phenomenon. More importantly, this ZBA splitting can be turned on and off alternately by changing the electron occupancy within the QDs since the two-impurity Kondo system requires both QDs to contain an odd number of electrons, i.e., two spatially separate non-zero spins[1–3]. Accordingly, QD Kondo state can be detected by operating occupancy of a coupled ER with various reported structures from nearly-closed QDs to Fabry–Pérot interferometers[5,12]. An unpaired ER spin causes a ZBA splitting in the studied QD while the ER charge states with zero spin only modulates the ZBA peak height.

For QPC electrons, a phase shift due to the Kondo state has previously been observed using scanning gate microscopy[13]. When a biased gate probe approaches the QPC, alternating single- and double-peak ZBAs emerge as a result of changes in the QPC electrostatic potential[14]. Similarly, in conventional QPCs formed by a pair of split gates, either single- or double-peak ZBAs can

appear when biased at different conductance values[15]. This behavior has been further clarified by the observation of alternating single- and double-peak ZBAs in a length-tunable QPC composed of multiple closely spaced pairs of split gates[15]. In such a system, the oscillatory 0.7-anomaly conductance is coupled to ZBA peak parity switches as the channel length is modulated, making the two effects indistinguishable. Moreover, the QPC quasi-bound state has been identified by analyzing path interference between QPC electrons, both with and without involving a quasi-bound state in another spatially separate QPC[16–18]. In this seminal work, the quasi-bound charge state was detected only at pinch-off (i.e., when G vanishes) and moreover no information was provided about the spin texture. Although various studies on ZBA behaviors and electron phase shifts suggest the existence of a QPC Kondo spin, its microscopic origin and dynamics remain unclear. This is because tuning the QPC channel potential or conductance affects not only the parity of quasi-bound spins but also the van-Hove-ridge scenario and other coexisting non-Kondo mechanisms. Additionally, in a multi-gate device, interaction-driven quasi-bound states may form in nanoconstrictions created by each pair of gates. Therefore, to better reveal QPC Kondo physics, the number of biased gates should be minimized as much as possible, as implemented in device A.

Supporting Note 3. Supporting data and analyses from devices T1 and T2

Figures S1a-S1d show the conductance data obtained from the test device T1 (with the gate pattern shown in the inset of Fig. S1a). The gate voltages $V_{1\text{-}4}$ and $V_{5,6}$ are applied to form the quantum dot (QD) and quantum point contact (QPC) constrictions, respectively. With tuning V_2, the QD conductance G_{QD} shows Coulomb blockade oscillations due to successive changes in the QD electron occupancy (Figs. S1a and S1b). The QD transmission is different at two different gate voltage settings for V_1, V_3, and V_4), giving rise to two different fingerprints of Coulomb blockade oscillations.

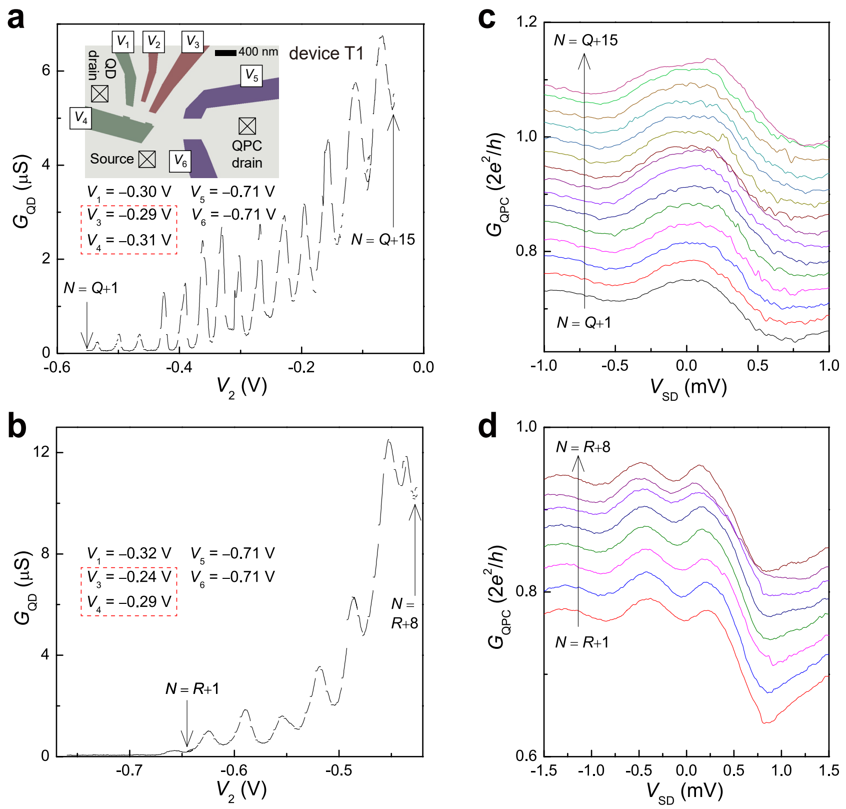

Figure S1: **Odd-even Kondo-spin-parity control in device T1.** (**a, b**) Lithographic gate pattern with the applied voltages is shown in the inset of (**a**). Conductances G_{QD} for the quantum dot (QD, formed by applying the gate voltages $V_{1\text{-}4}$) and G_{QPC} for the quantum point contact (QPC, formed by $V_{5,6}$) were simultaneously measured at two different gate voltage settings. The integer N denotes the electron number in the QD. Coulomb blockade spectra in G_{QD} due to successive changes in the QD electron occupancy from $N = Q + 1$ to $N = Q + 15$ and from $N = R + 1$ to $N = R + 8$ with V_2 are resolved in (**a**) and (**b**), respectively. (**c, d**) G_{QPC} against the source-drain bias V_{sd} in (**c**) and (**d**) were measured under different QD electron occupancies in different Coulomb blockade valleys via the control of V_2 (increasing along the arrow direction) as denoted in (**a**) and (**b**), respectively. Traces are offset by steps of 2 μS from bottom for clarity.

The rising QD conductance background with V_2 results from the simultaneous V_2 control over the QD electron occupancy and potential barrier. The QPC constriction is tuned to be in the tunneling regime with the conductance $G_{\text{QPC}} < G_{\text{Q}} = 2e^2/h$. We measured the QPC conductance against the source-drain bias V_{sd} (i.e., the nonlinear conductance trace) at a sequence of specific V_2 (leading to the Coulomb valleys in Figs. S1a and S1b, indicated by arrows) to track the evolution of the QPC ZBA with successively changing the QD electron number (see Figs. S1c and S1d, respectively). The ZBA in the nonlinear conductance exhibits a peak splitting with increasing V_2 (Fig. S1c) and becomes more obvious in its two-peak character with lowering the right QD potential barrier (close to the QPC) via $V_{3,4}$ (Fig. S1d).

Figure S2a shows the Coulomb blockade spectrum of the QD while Fig. S2b shows the QPC nonlinear conductance traces with successively tuning the QD electron occupancy in device T2 (with the gate pattern shown in the inset of Fig. S2a). We have modified the spatial arrangement of QD and QPC to reduce the influence of V5 (required to form the QPC) on the QD right barrier and demonstrated that V_2 can now control not only the QD electron occupancy but also the ZBA peak parity (indicated by triangles in Fig. S2b). With increasing V_2, two switches between single- and double-peak ZBAs were observed. Data from both devices T1 and T2 show that the observed alternating single- and double-peak ZBAs are not related to the QD occupancy parity. Instead, the state occupancy of an accidental electronic resonator (ER), formed between the QPC and QD, plays a key role in controlling the QPC Kondo state and ZBA peak parity. This explains the ER-QPC coupling mechanism in our study.

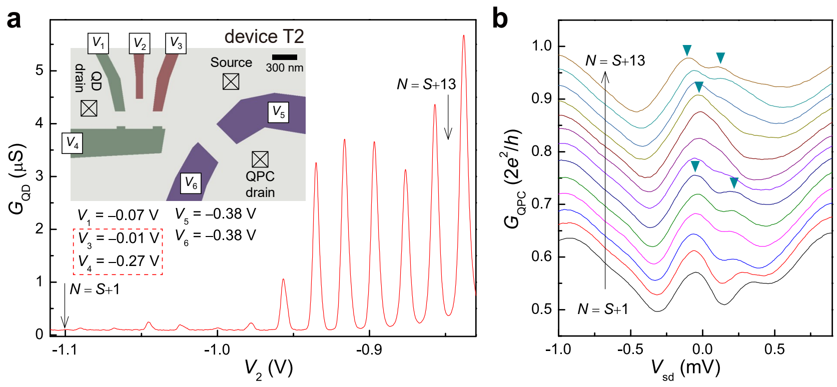

Figure S2: **Odd-even Kondo-spin-parity control in device T2.** (**a**) QD conductance G_{QD} as a function of the plunger gate voltage V_2 and thereby the electron occupancy. Inset, lithographic gate pattern with the applied gate voltages. (**b**) Simultaneously measured QPC conductance G_{QPC} against V_{sd} with tuning the QD electron occupancy from $N = S + 1$ to $N = S + 13$, where S is an integer, via V_2 control as denoted in (**a**). Traces are offset by steps of 2 μS from bottom for clarity. Triangles, indicating the zero-bias anomaly (ZBA) conductance peaks, in (**c**) highlight the switches between single- and double-peak ZBAs with tuning V_2.

Supporting Note 4. Extended data and analyses for device A

Our observed temperature T-dependent and magnetic field B-dependent ZBAs are inconsistent with the prediction for a QD two-impurity Kondo system[1], which we attribute to the influence of other coexisting QPC states. Firstly, by the conventional methods to estimate the characteristic Kondo temperature T_{K} (below which the Kondo-type ZBA occurs) using the QD and empirical QPC models[19], we obtain $T_{\mathrm{K,QD(QPC)}} \approx 13$ K and 9 K, respectively, which are much higher than $T_{\mathrm{ZBA}} = 1.1$ K (at which the ZBA is suppressed) in our system (see Fig. S3b). Secondly, if the merging of the two ZBA peaks in the two-impurity Kondo state were completely due to the Zeeman effect compensating for the spin singlet gap, further increasing B should also cause the ZBA to split. In our experiments, the ZBA peaks in the single- and two-impurity Kondo states do not split or re-split but merely collapse with B.

The T dependence of zero-bias G show a deviation from the prediction of Kondo model for $T > 1$ K (see Fig. S3b). We find that the thermal depopulation of a QPC subband with the characteristic temperature $T_{\mathrm{A}} \approx 1$ K can give a reasonable fitting result and explain the non-Kondo behavior for $T > 1$ K (see Fig. S3b, inset). It was reported that the QPC subband, contributing to such a temperature dependence, is spin-split due to many-body interactions[20–23]. Moreover, the zero-bias G data for different ZBA parities are also well fitted to the quadratic curves for 0.5 K $< T < 1$ K on the basis of the van Hove ridge mechanism[24,25], as shown in Fig. S4a. It indicates that the Kondo effect and van Hove ridge may coexist and they cannot be simply distinguished in this temperature range. For $T < 0.5$ K, the Kondo mechanism dominates so that the conductance can reveal either single- or double-peak ZBA in response to the ER occupancy parity. This explains the observed disappearance of double-peak ZBA at $T > 0.6$ K by the significant influence of

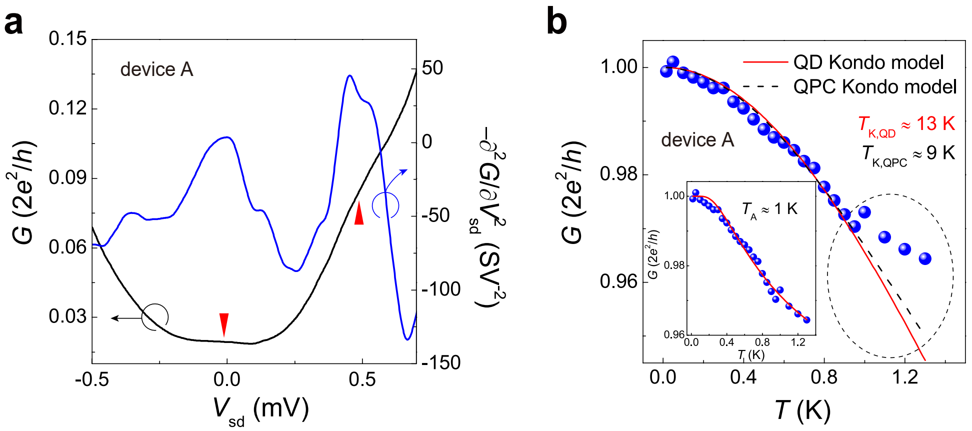

Figure S3: **Two-impurity Kondo state at low conductance and Kondo temperature estimate for device A.** (**a**) The second derivative of the nonlinear conductance at $V_{\rm er} = -2$ V and $V_{\rm qpc} = -1.6$ V. Data at other $V_{\rm er}$ with the same $V_{\rm qpc}$ are given in Fig. 3b, main article. Plotting the second derivative allows the zero-bias-anomaly (ZBA) to be identified even at very low conductance (triangles). (**b**) The single-impurity ZBA peak conductance as a function of temperature from the data in Fig. 4b, main article. The solid curves correspond to fits below 1 K and its extension using the quantum dot model $\frac{2e^2}{h}(\frac{T'^2_{\rm K,QD}}{T^2+T'^2_{\rm K,QD}})^s$ and empirical quantum point contact model $\frac{2e^2}{h}[1/2(\frac{T'^2_{\rm K,QPC}}{T^2+T'^2_{\rm K,QPC}})^s + 1/2]$, where $T'_{\rm K,QD(QPC)} = T_{\rm K,QD(QPC)}/\sqrt{2^{1/s}-1}$ and $s = 0.2$ with e, h, and $T_{\rm K,QD(QPC)}$ being the electron charge, Planck's constant, and Kondo temperature, respectively[19,26]. Inset, a fit to the activation model $\frac{2e^2}{h}(1 - C\exp(-T_{\rm A}/T))$, where $T_{\rm A}$ is the activation temperature and C is the fitting parameter[27].

the van Hove ridge (Fig. 4a, dashed trace). For $T > 1$ K, both the Kondo and van Hove ridge models fail to fit the data due to the thermal depopulation of a QPC subband. The coexistence of Kondo effect and van Hove ridge also plays a role in the B dependence of zero-bias G (see Fig. S4b). For $B > 3$ T, a quadratic B dependence appears due to the influence of van Hove ridge[24,25]. At low fields $B < 3$ T, the deviation from the quadratic B dependence in zero-bias G and the observation of ZBA parity change in nonlinear G indicate the predominance of Kondo effect. Moreover, the ZBA peak splitting exhibits a nonlinear dependence on B, suggesting that the Zeeman effect—expected to produce a linear B dependence in peak splitting—is not the sole factor responsible for the B-induced merging of the two ZBA peaks in the two-impurity Kondo state. Thus, we cannot expect the peak re-splitting at $B = 5$ T to have the same magnitude as at $B = 0$, given the presence of various coexisting QPC states. These results further highlight the intricate electronic states in a high-mobility quasi-1D nanoconstriction.

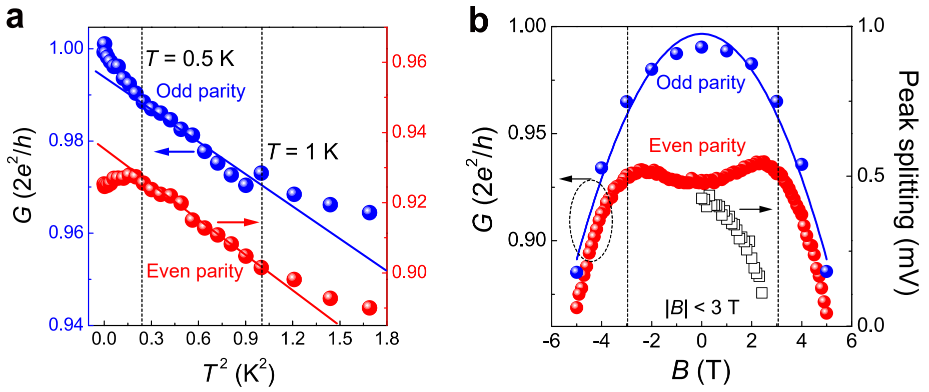

Figure S4: **Coexistence of QPC states in device A.** (**a**) Conductance data obtained from Figs. 4a and 4b at $V_{\mathrm{sd}} = 0$ and 0.05 mV (main article) for single-impurity (odd-parity) and two-impurity (even-parity) Kondo states. Solid lines are guides to the eye, showing the quadratic dependences. (**b**) Conductance data obtained from Figs. 4c and 4d at $V_{\mathrm{sd}} = 0$ and 0.05 mV (main article) for odd- and even-parity Kondo states, respectively (left axis). Solid curves are guides to the eye, showing the quadratic dependence. The peak splitting in the double-peak ZBA with B is also presented (right axis).

References

1. H. Jeong, A. M. Chang & M. R. Melloch, The Kondo effect in an artificial quantum dot molecule. *Science* **293**, 2221 (2001).

2. N. J. Craig *et al.*, Tunable nonlocal spin control in a coupled-quantum dot system. *Science* **304**, 565–567 (2004).

3. J. C. Chen, A. M. Chang & M. R. Melloch, Transition between quantum states in a parallel-coupled double quantum dot. *Phys. Rev. Lett.* **92**, 176801 (2004).

4. P. Simon, R. López & Y. Oreg, Ruderman-Kittel-Kasuya-Yosida and magnetic-field interactions in coupled Kondo quantum dots. *Phys. Rev. Lett.* **94**, 86602 (2005).

5. R. M. Potok, I. G. Rau, H. Shtrikman, Y. Oreg & D. Goldhaber-Gordon, Observation of the two-channel Kondo effect. *Nature* **446**, 167 (2007).

6. A. Keller *et al.*, Universal Fermi liquid crossover and quantum criticality in a mesoscopic system. *Nature* **526**, 237–240 (2015).

7. J. Bork*et al.*, A tunable two-impurity Kondo system in an atomic point contact. *Nat. Physics* **7**, 901–906 (2011).

8. H. Prüser *et al.*, Interplay between the Kondo effect and the Ruderman–Kittel–Kasuya–Yosida interaction. *Nat. Commun.* **5**, 5417 (2014).

9. T. Esat *et al.*, A chemically driven quantum phase transition in a two-molecule Kondo system. *Nat. Physics* **12**, 867–873 (2016).

10. C. Duan *et al.*, Incommensurate spin fluctuations in the spin-triplet superconductor candidate UTe_2. *Phys. Rev. Lett.* **125**, 237003 (2020).

11. S. S. Yeh *et al.*, Oxygen vacancy-driven orbital multichannel Kondo effect in Dirac nodal line metals IrO_2 and RuO_2. *Nat. Commun.* **11**, 4749 (2020).

12. C. Rössler *et al.*, Transport spectroscopy of a spin-coherent dot-cavity system. *Phys. Rev. Lett.* **115**, 166603 (2015).

13. B. Brun *et al.*, Electron Phase Shift at the Zero-Bias Anomaly of Quantum Point Contacts. *Phys. Rev. Lett.* **116**, 136801 (2016).

14. B. Brun *et al.*, Wigner and Kondo physics in quantum point contacts revealed by scanning gate microscopy. *Nat. Commun.* **5**, 4290 (2014).

15. M. J. Iqbal *et al.*, Odd and even Kondo effects from emergent localization in quantum point contacts. *Nature* **501**, 79 (2013).

16. Y. Yoon *et al.*, Detector backaction on the self-consistent bound state in quantum point contacts. *Phys. Rev. B* **79**, 121304 (2009).

17. Y. Yoon *et al.*, Coupling quantum states through a continuum: a mesoscopic multistate Fano resonance. *Phys. Rev. X* **2**, 021003 (2012).

18. J. Fransson *et al.*, Tuning the Fano resonance with an intruder continuum. *Nano Lett.* **14**, 788 (2014).

19. S. M. Cronenwett *et al.*, Low-temperature fate of the 0.7 Structure in a point contact: A Kondo-like correlated state in an open system. *Phys. Rev. Lett.* **88**, 226805 (2002).

20. K. J. Thomas *et al.*, Possible spin polarization in a one-dimensional electron gas. *Phys. Rev. Lett.* **77**, 135–138 (1996).

21. A. Kristensen *et al.*, Bias and temperature dependence of the 0.7 conductance anomaly in quantum point contacts. *Phys. Rev. B* **62**, 10950 (2000).

22. Y. Tokura & A. Khaetskii Towards a microscopic theory of the 0.7 anomaly. *Physica E* **12**, 711 (2002).

23. L. P. Rokhinson, L. N. Pfeiffer & K. W. West, Spontaneous spin polarization in quantum point contacts. *Phys. Rev. Lett.* **96**, 156602 (2006).

24. F. Bauer *et al.*, Microscopic origin of the '0.7-anomaly' in quantum point contacts. *Nature* **501**, 73 (2013).

25. D. H. Schimmel, B. Bruognolo & J. von Delft, Spin fluctuations in the 0.7 Anomaly in quantum point contacts. *Phys. Rev. Lett.* **119**, 196401 (2017).

26. D. Goldhaber-Gordon *et al.*, From the Kondo regime to the mixed-valence regime in a single-electron transistor. *Phys. Rev. Lett.* **81**, 5225–5228 (1998).

27. F. Sfigakis *et al.*, Kondo effect from a tunable bound state within a quantum wire. *Phys. Rev. Lett.* **100**, 026807 (2008).
